# Data of bacterial community dynamics resulting from total rumen content exchange in beef cattle

**DOI:** 10.1186/s13104-021-05726-1

**Published:** 2021-08-10

**Authors:** Brooke A. Clemmons, Madison T. Henniger, Phillip R. Myer

**Affiliations:** 1 Department of Animal Science, University of Tennessee, Knoxville, TN 37996 USA; 2 Present Address: Department of Agriculture, Texas A&M University-Commerce, Commerce, TX 75428 USA

**Keywords:** Rumen, Bacteria, Microbiome, Host–microbiome interactions

## Abstract

**Objectives:**

Extensive efforts have been made to characterize the rumen microbiome under various conditions. However, few studies have addressed the long-term impacts of ruminal microbiome dysbiosis and the extent of host control over microbiome stability. These data can also inform host-microbial symbioses. The objective was to develop preliminary data to measure the changes that occur in the rumen bacterial communities following a rumen content exchange to understand the effects major perturbations may impart upon the rumen microbiome, which may be host-driven.

**Data description:**

We report here an initial rumen content exchange between two SimAngus (Simmental/Angus) non-pregnant, non-lactating cows of ~ 6 years of age weighing 603.4 ± 37.5 kg. To measure bacterial community succession and acclimation following the exchange, rumen content was collected via rumen cannula at the beginning of the study immediately prior to and following the rumen content exchange, and weekly for 12 weeks. The V4 hypervariable region of the 16S rRNA gene was targeted for DNA sequencing and bacterial analysis. Over 12 weeks, numerous genera and diversity varied, before partial return to pre-exchange metrics. These preliminary data help support potential host control for the rumen microbiome, aiding in efforts to define bovine host-microbe relationships.

## Objective

Cattle genetics accounts for a wide range of phenotypes resulting in important production parameters, including milk production, carcass quality, and feed efficiency [1–3]. However, ruminants possess a unique rumen microbiome that contributes to the successful conversion of low quality feedstuffs to high-quality protein and has also been linked to feed efficiency, methane production, and other critical production traits [4–6]. Not surprisingly, diet plays a main role in determining the composition of the gut microbiota. Several studies have also shown that the rumen microbiota vary according to the breed of the ruminant host [7–9], which suggests that genetic variation in the host may also influence the composition of the microbiome. Indeed, recent data have begun to demonstrate that host genetics may help guide the establishment of microbiota in the gut of ruminants and other animals [1, 8, 10]. Previous studies have demonstrated the cross-inoculation of ruminal contents between pairs of cows may indicate host–microbe interactions through the near-complete return to pre-exchange ruminal conditions [11–13]. Yet, these studies were almost completely focused on dairy cattle and dairy production. Although the importance of the rumen microbiome is well established in beef cattle [14, 15], the host influence on the composition and function of the rumen bacterial communities is poorly understood. This gap in knowledge is critical to fill because it presents the opportunity to enhance feed efficiency, disease resistance, and other production traits that are important to improve to meet the growing need for global food production and security [14]. Therefore, these preliminary data were collected to examine the beef cattle host specificity of the ruminal bacterial community. Ultimately, these data were utilized to help discern the ability of the rumen bacterial community to reestablish following a major rumen perturbation.

## Data description

The rumen bacterial community data acquired were the result of a near-total rumen content exchange (RCE). To obtain these data, two previously ruminally cannulated SimAngus (Simmental/Angus) non-pregnant, non-lactating, healthy cows of ~ 6 years of age weighing 603.4 ± 37.5 kg were sourced from the Plateau AgResearch and Education Center in Crossville, TN and placed on a primarily corn silage-based diet (11.57% crude protein and 76.93% total digestible nutrients on a dry matter basis) and acclimated to the diet for a two-week period in a GrowSafe system. Rumen content was collected via rumen cannula at the beginning of the study immediately prior to the RCE. Gaining access from the rumen cannula, the ruminal contents were almost completely removed, stored in a container, and transferred/redeposited manually between cows similar to previous methods [11, 12]. Rumen content was then collected immediately following the RCE, and weekly for 12 weeks for a total of 26 samples. To analyze the changes in bacterial community composition to determine the potential and extent of host reestablishment of bacterial communities in the rumen, the V4 hypervariable region of the bacterial 16S rRNA gene was targeted for 16S-based amplicon DNA sequencing on the Illumina MiSeq for bacterial community identification and analyses.

The resulting data consisted of DNA base call and quality files (FASTQ; Table 1, Data Set 1). The raw data were analyzed in the R environment and using the mothur software package (v. 1.39.5) [16]. The raw data were processed to determine the taxonomic profiles and diversity of the bacterial communities prior to and following RCE. Although these were preliminary data, it was critical to analyze the raw data to this extent to determine the potential and extent of bacterial community reestablishment in the rumen following a major rumen perturbation.

**Table 1:** Overview of data files/data sets

Label	Name of data file/data set	File types (file extension)	Data repository and identifier (DOI or accession number)
Data Set 1	Raw DNA Reads Rumen Content Exchange	FASTQ (.fastq)	NCBI Sequence Read Archive (https://www.ncbi.nlm.nih.gov/bioproject/PRJNA704677/) [19]
Data File 1	NMDS RCE	TIFF (.tiff)	Figshare (https://doi.org/10.6084/m9.figshare.14114168.v1) [20]
Data File 2	Genus-level proportions of bacterial communities	TIFF (.tiff)	Figshare (https://doi.org/10.6084/m9.figshare.14114219.v2) [21]
Data File 3	Methodology	PDF (.pdf)	Figshare (https://doi.org/10.6084/m9.figshare.14191868.v3) [22]

Beta diversity was visualized using a non-metric multidimensional scaling (NMDS) plot, informed by the raw data and Bray–Curtis indices, and was produced and depicted in Data File 1. An NMDS is an approach which produces an ordination based on a distance or dissimilarity matrix. The NMDS in Data File 1 depicts the community composition similarities between samples as a function of week and pre- or post-RCE. Following RCE, although the rumen communities between cows did not completely restore to those of the pre-exchange communities of the donor communities, they generally moved toward reestablishment of their pre-exchange communities by week 12 following RCE.

The raw data were also denoised and taxonomically classified using the SILVA v132 database [17] as the reference database. Barplots were generated depicting the relative abundances of the most abundant genera prior to and following RCE between cows for 12 weeks (Data File 2). Approximately 30% of the genera belonged to species of the genus *Prevotella*, followed by the commonly abundant ruminal genera of *Succiniclasticum* and *Ruminococcus* [18]. Cow 1291 had greater populations of the family Succinivibrionaceae than Cow 1651 pre-exchange, which similar differential abundances were appropriately detected in Cow 1651 following RCE and in week 1. However, by week 12, the populations of Succinivibrionaceae drastically reduced, indicating general reestablishment of pre-exchange communities.

## Limitations

The data were collected as part of a preliminary study to examine resilience of the rumen bacterial community to reestablish following a major rumen perturbation as a means to demonstrate the beef cattle host specificity of the ruminal bacterial community. These data were derived from an experiment utilizing two cows. A few studies in ruminally fistulated Holstein cows have utilized this method and approach with an equivalent or small sample size and statistical power [11]. However, limitations in the current dataset still do exist due to the lack of statistical power, but the results were significant from multiple testing among days. Regardless of the lack of power, this preliminary information is helpful to describe the ongoing work relating to microbial community reestablishment in the rumen following a major rumen perturbation. The data are also representative of the specific herd microbial community and its resilience, as these animals originated from the same herd and location.

## Data Availability

The data described in this Data note can be freely and openly accessed on Figshare or the National Center for Biotechnology Information Sequence Read Archive under accession number PRJNA704677. Please see Table 1 and references [19–22] for details and links to the data.

## Abbreviations

NMDS: Non-metric multidimensional scaling
RCE: Rumen content exchange
